# The roles and values of wild foods in agricultural systems

**DOI:** 10.1098/rstb.2010.0123

**Published:** 2010-09-27

**Authors:** Zareen Bharucha, Jules Pretty

**Affiliations:** Interdisciplinary Centre for Environment and Society and Department of Biological Sciences, University of Essex, Colchester, Essex, UK

**Keywords:** wild foods, hunters and gatherers, conservation, ecosystem services

## Abstract

Almost every ecosystem has been amended so that plants and animals can be used as food, fibre, fodder, medicines, traps and weapons. Historically, wild plants and animals were sole dietary components for hunter–gatherer and forager cultures. Today, they remain key to many agricultural communities. The mean use of wild foods by agricultural and forager communities in 22 countries of Asia and Africa (36 studies) is 90–100 species per location. Aggregate country estimates can reach 300–800 species (e.g. India, Ethiopia, Kenya). The mean use of wild species is 120 per community for indigenous communities in both industrialized and developing countries. Many of these wild foods are actively managed, suggesting there is a false dichotomy around ideas of the agricultural and the wild: hunter–gatherers and foragers farm and manage their environments, and cultivators use many wild plants and animals. Yet, provision of and access to these sources of food may be declining as natural habitats come under increasing pressure from development, conservation-exclusions and agricultural expansion. Despite their value, wild foods are excluded from official statistics on economic values of natural resources. It is clear that wild plants and animals continue to form a significant proportion of the global food basket, and while a variety of social and ecological drivers are acting to reduce wild food use, their importance may be set to grow as pressures on agricultural productivity increase.

## 1. Introduction

‘Any bloke hungry in this country just silly’

(Yarralin man, Northern Territory, Australia: in Bird Rose 1996), p. 99

‘What do you mean by weeds? There is nothing like a weed in our agriculture’

(Woman farmer, Deccan plateau, India: in Mazhar *et al*. 2007), p. 18.

Globally, an estimated 1.02 billion people are undernourished (FAO 2009). The literature on vulnerability, food security and ecosystem services has tended to emphasize cultivated foods (MEA 2005; Ericksen *et al*. 2009). However, there is substantial evidence that wild foods are an important part of the global food basket. At regional and national level, food balances guide policies on trade, aid and the declaration of food crises. Notably absent from these is the contribution made by wild edible species. With the routine underestimation of wild foods comes the danger of neglecting the provisioning ecosystems and supportive local knowledge systems that sustain these food chains (Grivetti & Ogle 2000; Mazhar *et al*. 2007; Pilgrim *et al*. 2008).

We summarize the best available evidence for the importance and values of wild foods (see Scoones *et al*. 1992; Heywood 1999; Posey 1999; MEA 2005; Kuhnlein *et al*. 2009).

## 2. Hunter–gatherers, foragers, fishers and cultivators

### (a) Stereotypes

A central assumption about non-agricultural societies has been that they represent an earlier stage of cultural evolution, or the outcome of cultural devolution (Barnard 1999). It was long supposed that cultures progressed from hunter–gatherer to agricultural to industrial. Beginning with Hobbes's 1651 observation that the life of ‘natural man’ was ‘solitary, poore, nasty, brutish and short’, cultural evolutionary views—distinguishing between ‘natural’ and ‘civilized’ peoples—persisted from the eighteenth to the late twentieth centuries (Meggers 1954; Lathrap 1968). Lathrap, for example, uses terms such as devolution, degradation and wreckage of former agricultural societies to describe communities in the Amazon that engage in hunting, gathering and foraging (Barnard 1999).

Evidence has revealed the limitations of these perspectives (Kent 1989; Kelly 1995). The landmark *Man the Hunter* conference and book (Lee & DeVore 1968) showed hunter–gatherers to be rich, knowledgeable, sophisticated and above all different from one another. There was no single stage of human development, just different adaptations to ecological and social circumstances. It is now better accepted, though not universally, that cultures are adapted to localities, and thus are configured with a wide variety of land uses and livelihoods. As a result, foraging and farming across the world are actually ‘overlapping, interdependent, contemporaneous, coequal and complementary’ (Sponsel 1989). This suggests that many rural people and their cultures might be better known as variants of cultivator–hunters or farmer–foragers rather than just farmers or hunter–gatherers. Culture and nature are thus bound together (Berkes 1999; Pretty *et al*. 2010).

Another long-standing stereotype suggests that hunter–gatherers are nomadic and cultivators sedentary. Again, the evidence shows a bewildering array of adaptations and cultural choices. Some horticulturalists move, some hunter–gatherers are sedentary (Vickers 1989; Kelly 1995). Some groups maintain gardens for cultivated food as well as to attract antelopes, monkeys and birds for hunting (Posey 1985). Many apparently hunter–gatherer and forager cultures farm; many agricultural communities use large numbers of non-domesticated resources. The Hohokam are well-known as sophisticated canal irrigators and desert farmers of the American southwest, yet they were hunters, gatherers and foragers too. Szuter & Bayham (1989) thus observed that the ‘convenient labels of hunter–gatherer or farmer are of minimal value… The two activities are complementary’.

### (b) The management of non-agricultural environments

What has also become clear is that farmers, hunters, gatherers, fishers and foragers do not simply take resources from a compliant environment. They manage and amend resources in much the same way as is standard practice on farms (table 1). Foragers maintain resources by intentional sowing of wild seeds, irrigation of stands of grasses, burning to stimulate plant growth, selective culling of game animals and fish, replanting of portions of roots, enrichment planting of trees and extraction of only parts of honeycombs so that sites are not deserted by bees (Steward 1938; Lawton *et al*. 1976; Woodburn 1980; Kelly 1995). All these activities have agricultural equivalents, and are variously designed to increase the productivity and stability of useful plants and animals.

**Table 1.** The management of non-agricultural ecosystems by farmers, hunter–gatherers and foragers. Sources: Kent (1989), Rosman & Rubel (1989), Kelly (1995), Bird Rose (1996), Balée (1998), Fowler & Turner (1999), Kehoe (1999), Pretty (2002, 2007), Harris & Mohammed (2003), Anderson & Nuttall (2004), Berkes (1999, 2009), Brookfield & Padoch (2007), Stephenson & Möller (2009) and Heckenberger (2009).

practice	detail	agricultural equivalent	examples
harvesting and hunting	hunting of particular species or individuals, at particular times	crop harvesting	muttonbird (sooty shearwater) gathering by Maori
	sparing young animals and fish	livestock raising	aboriginal caretakers
	rotational hunting and no-take zones closed fishing areas and closed		beaver bosses of Cree, rested hunting and trapping areas
	season allowing portion of fish catch to escape		sparing lead caribou individuals (as have knowledge of migration routes)
	taboos and rituals for certain people and animals		Pacific island closed fishing areas and seasons
	nomination of stewards to regulate hunting		
planting	enrichment planting of fruit and medicinal trees in forests and home gardens	planting of domesticated seeds	tree, palm and bamboo enrichment by Amazonian cultures aboriginal wild gardens
	scattering seeds and roots		distribution and reproduction of
	replacing portions of roots		mongongo nut trees by San
	replanting of propagules		transplanting willow for basketry by
	selectively tended wild gardens		Shoshone
	agroforestry on and off farms		
raising animals	selective culling and sparing	raising domesticated	managing wild pigs in Papua New
	transplanting eggs and young	animals	Guinea
	feeding young animals		
nutrient additions	human and animal wastes near settlements mulching and charcoal as soil amendments	fertilizer, compost, animal manure	pastoralist corrals in Sub-Saharan Africa leading to Acacia woodlands wild pig management in Papua New
	feed for fish and wild pigs		Guinea
pest management	protection by removal of weeds, pests or predators	pest management	management of oyster beds in UK
habitat amendment and creation	coppicing and thinning of trees to increase yields and biodiversity	habitat amendment for agriculture	swidden agriculturalistsfarmers creating ponds for fishing or
	creation of ponds and fleets		wildfowling in UK
	creation of maize and sorghum game cover		farmers maintaining woodland and game cover for shooting
	clearing of forest glades		
	creation of rock cairns to attract lizards		
	creation of hunting gardens		
water management	diversion of streams to irrigate wild strands of grasses	irrigationdrainage	irrigation by Hohokam in USA
	channel diversion for fish trapping		
	clearing of stream-beds for fish spawning		
fire use	burning to increase grass yields to encourage game, reveal burrows and tracks	burning crop stubbles and strawclearing swiddens	firestick farming by Australian Aboriginescreation of parklands by Native
	broadcasting seeds of annuals and perennials after burning	burning heather moors	Americans (Yosemite and Vancouver Island)
			burning of prairies by Blackfoot to improve grasses for wild herds

Many cultures and groups directly manage trees on and off the farm. The forest islands of Amazonia were found by Posey (1985) to have emerged as a result of Kayapo directly planting-up mounds. In the lower Amazon, smallholder farmers enrich the forests with desirable fruit, timber and medicinal trees, often broadcasting seeds when cutting timber (Brookfield & Padoch 2007). In dryland Kenya, *Acacia tortilis* tree recruitment occurs on the sites of abandoned pastoralist corrals that are high in organic matter and nutrients from the penned livestock. Acacia seedpods are a favoured fodder, and some pass through the animals to then germinate in the next season. The result is circular woodlands of dense Acacia (Reid & Ellis 1995; Berkes 1999). In China, there is widespread use of wild trees in integrated systems of land management, and wild plants and animals are gathered from a variety of microenvironments, such as dykes, woods, ponds and irrigation ditches (Li Wenhua 2001).

Farmers also widely transplant species from the wild. In northern Nigeria, they plant *Hibiscus* on field boundaries; in South Africa, wild fruit trees and edible herbs are grown on farms; and in northeastern Thailand, a quarter of all the 159 wild food species gathered from field boundaries, irrigation canals, swamps and roadsides are transplanted and propagated by rice farmers (Price 1997; High & Shackleton 2000; Harris & Mohammed 2003). Home gardens are particularly important for many rural smallholders, and are notably diverse, sometimes containing more than 200 useful species (Eyzaguirre & Linares 2004). In northeast Thailand, 88 per cent of home gardens contain wild species. Home gardens are often a refuge for wild species threatened by deforestation and urbanization, and in periods of drought when the wild relatives suffer, those surviving in the home gardens provide considerable additional value to farm households.

Burning is a widespread management practice. Australian Aborigines call it ‘firestick farming’, and used fire to make the ‘country happy’, to keep it ‘clean’ (Bird Rose 1996). Burning allowed people to walk without fear of snakes and the nuisance of grass seeds; it created new food for kangaroos and wallabies; and made it easy to see animal tracks and burrows. The observation of smoke is still taken to be a sign that the country is healthy. Burning was also common in North America, helping to create the ‘parkland’ type environments of Yosemite and Vancouver Island, and used by plains groups to increase herd size on the prairies (Berkes 1999; Lee & Daly 1999).

To many cultures, the ideas of wild, wildlife and wilderness remain problematic. The term wild is commonly used today to refer to ecosystems and situations where people have not interfered, yet we now know that people influence, interfere with and manage most if not all ecosystems and their plants and animals. In Papua New Guinea, wild and domesticated pigs are central to many subsistence strategies (Rosman & Rubel 1989). Wild pigs are hunted and managed: boars and sows are brought together to breed, females are followed to their nests, litters and piglets removed for raising, and wild pigs are fed with sago and roots. Some groups raise extra gardens of sweet potatoes just for pigs. Forest-dwelling cassowaries are never bred, but their chicks are captured, tamed and raised. Similar merging of the wild and raised occurs in reindeer (caribou) herding and hunting communities of Siberia (e.g. Evenki, Anderson 1999).

What is common in all cases is that people pay close attention to what the land is telling them. Such knowledge and understanding is then encoded into norms, rules, institutions and stories, and thus forms the basis for continued adaptive management over generations (Basso 1996; Pretty 2007; Berkes 2009). This knowledge is an important capital resource. The result is a huge variety of subsistence strategies that vary spatially as well as over time (Kelly 1995).

### (c) Farmers and wild foods

In both agricultural and hunter–gatherer systems, there are no easy distinctions between ‘wild’ and ‘cultivated’ foods. While food research and policy tend to consider these separately, the differences are rarely mirrored by local communities. Plant foods can thus be envisioned as ‘existing along a continuum ranging from the entirely wild to the semi-domesticated, or from no noticeable human intervention to selective harvesting, transplanting, and propagation by seed and graft’ (Harris 1989). Moreover, since ‘domestication grew out of food gathering, which almost imperceptibly led to cultivation’, many wild edible species can be considered to be ‘in various stages of domestication as a result of human selection, however slight’ (Heywood 1999). Many farmers continually blur the distinction between the cultivated and the uncultivated (Mazhar *et al*. 2007).

Wild foods have long provided farmers a ‘hidden harvest’, as they have used co-evolved species and other wild biodiversity in and around their farms to supplement their foods and earnings (Harris & Hillman 1989; Scoones *et al*. 1992; Heywood 1999; Grivetti & Ogle 2000). Many species are found within the fields themselves. The harvesting of wild species from paddy fields is an excellent example; in Thailand, farmers harvest wild herbs, insects, trees and vines (Price 1997; Halwart 2008); in Bangladesh, 102 species of greens and 69 of fish (Mazhar *et al*. 2007) are collected. In Svay Rieng, Cambodia, wild fish from in and around paddies contribute up to 70 per cent of total protein intake as well as being a source of income. Their relevance as a buffer against hunger is considerable in this area since rice yields here are among the lowest in southeast Asia (Guttman 1999). Table 2 summarizes the range of species used by rice-based agricultural communities in four Asian countries, with total use varying from 51 to 102 species (overall mean: 83; plants: 17; animals: 66).

**Table 2.** The diversity of aquatic wild food species within rice agroecosystems in four Asian contexts (adapted from Halwart 2008).

	Cambodia	China	Laos	Vietnam
plants	13	20	20	15
amphibians	2	3	10	3
crustaceans	6	4	5	3
fishes	70	54	26	14
molluscs	1	5	8	7
reptiles	8	1	7	3
insects	2	—	16	6
total	102	87	92	51

Farmers also transplant species onto or near fields. In northeast Thailand, a quarter of the 159 wild food species gathered are deliberately propagated (Price 1997; High & Shackleton 2000; Harris & Mohammed 2003). Smallholders' home gardens are another example—these are notably diverse, sometimes containing more than 200 useful species (Eyzaguirre & Linares 2004).

Wild food species are declining in many agricultural landscapes (MEA 2005). The spread of agriculture and the homogenization of agricultural landscapes increasingly limits the availability and use of wild foods of nutritional importance to agricultural communities, but most of all to the landless poor and other vulnerable groups (Scoones *et al*. 1992; Pretty 2002). Their continued availability depends on the maintenance of synergies between farming and wild biodiversity (Pretty 2007; Royal Society 2009).

## 3. The importance of wild foods

By FAO estimates, around ‘one billion people use wild foods in their diet’ (Aberoumand 2009). Forests provide livelihoods and food for some 300 million people in the form of non-timber forest products (NTFPs). In general, food security and NTFPs are strongly interlinked in rural communities, especially for the most vulnerable groups (Belcher *et al*. 2005), even among agricultural communities (Vincetti *et al*. 2008). Urban communities also rely on wild foods. For instance, affluent urban households are willing to pay 43–157% more for bushmeat in Zambia and Mozambique (Barnett 2000). In Rajasthan, India, wild foods benefit both urban and rural children (Rathore 2009). Titus *et al*. (2009) explored the importance of wild game in Alaska, where 80 per cent of the population is urban, and found urban households routinely consuming significant amounts of wild game.

### (a) The diversity of wild foods used

Food security has come to depend on a small handful of widely cultivated species. Over 50 per cent of the world's daily requirement of proteins and calories comes from three crops—wheat, maize and rice (Jaenicke & Höschle-Zeledon 2006); 12 species contribute 80 per cent of total dietary intake. By contrast, wild foods provide a greater dietary diversity to those who rely on them. Ethnobotanical surveys of wild plants indicate that more than 7000 species have been used for human food at some stage in human history (Grivetti & Ogle 2000; MEA 2005). Some indigenous communities use over 200 (Kuhnlein *et al*. 2009); in India, 600 plant species are known to have food value (Rathore 2009); DeFoliart (1992) records 1000 species of edible insects used worldwide. Some 1069 species of wild fungi consumed worldwide are important sources of protein and income (Boa 2004). Bushmeat and fish provide 20 per cent of protein in at least 60 developing countries (Bennet & Robinson 2000).

Additionally, wild plants in particular have diverse uses. In Nepal, 80 per cent of 62 wild food plants have multiple uses (Shrestha & Dhillon 2006). Tanzanian Batemi agro-pastoralists use species as food (31 species), thirst quenchers (six species), for chewing (seven species), as flavourants (two species) and for honey beer (one species). A further 35 wild edible plants are cultivated (Johns *et al*. 1996). In the Mekong Delta and Central Vietnamese Highlands, several wild food species are used as medicine and livestock feed; one-fifth are used as all three (Ogle *et al*. 2003).

We summarize evidence on the use of wild species in tables 3–5. Surveys of even small sample sizes yield surprisingly high numbers of species used. Table 3 illustrates the use of wild foods in 12 Asian contexts; table 4 in 10 countries across Africa. From these 36 studies in 22 countries of Asia and Africa, the mean use of wild foods (discounting country- or continent-wide aggregates) is 90–100 species per place and community group. Individual country estimates can reach 300–800 species (India, Ethiopia, Kenya). Table 5 illustrates the use of wild foods by 12 indigenous communities (seven agricultural; five hunter–gatherer) across both industrialized and developing countries. The mean use of wild species is 120 per community, rising to 194 for those seven communities formally designated as agricultural.

**Table 3.** The diversity of species of wild foods used in selected countries of Asia.

country	area characteristics	number of species	references
Bangladesh	floodplain rice farming communities	102	Mazhar *et al*. (2007)
Cambodia	rice field agroecosystem, lower Mekong basin	20	Shams *et al*. (undated)
Cambodia	rice field agroecosystem, Tonle Sap, Mekong basin	102	Balzer *et al*. (2003)
China	rice field agroecosystem in Xishuangbanna, Yunan Province	92	Halwart (2008)
India	general countrywide estimate	600	Rathore (2009)
India	tribal/non-tribal; cultivation and livestock, deciduous forest	73	Kala (2009)
India	tribal and non-tribal, transhumance and rainfed agriculture, temperate forests	21	Misra *et al*. (2008)
India	Mornaula Reserve Forest in western Himalaya	114	Pant & Samant (2006)
India	Sikkim Himalaya	190	Sundriyal & Sundriyal (2001)
India	rainfed agricultural community of Deccan Plateau; 79 species of plants used, plus hunting of monitor lizards, wild pigs, rabbits and fishes	79	Mazhar *et al*. (2007)
Jordan	arid, countrywide estimate	56	Tukan *et al*. (1998)
Lebanon	dry mediterranean, rural	6	Jeambey *et al*. (2009)
Mongolia	steppe, nomadic pastoralists	77	Huai & Pei (2000)
Nepal	rural, forest dwelling	62	Shrestha & Dhillon (2006)
Nepal	Chepang community, shifting cultivation	85	Aryal *et al*. (2009)
Palestinian Authority	rural agricultural communities (irrigated and rainfed) on West Bank	100	Ali-Shtayeh *et al*. (2008)
Thailand	irrigated rice in northeast and tropical/sub-tropical forest	159	Price (1997)
Thailand	Pwo Karen community; swidden cultivation in dry mixed deciduous forest	134	Delang (2006)
Turkey	western and central Anatolia	121	Dogan *et al*. (2004)
Vietnam	cultivation and livestock, Mekong Delta and Central Highlands	90	Ogle *et al*. (2003)

**Table 4.** The diversity of species of wild foods used in selected countries of Africa.

country	summarized area characteristics	number of species	references
Africa	continent-wide estimate (insects only)	600	DeFoliart (1992)
Africa	sub-Saharan Africa (insects only)	250	van Huis (2003)
Africa	Central and West Africa (plants only)	1500	Chege (1994)
Botswana	Tyua grow crops and use wild plants, animals, birds, fish and insects	171	Hitchcock (1999)
Congo	Mbuti Pygmies of forest: cultivators of cassava and plantain plus users of 230 animal and 100 plant species	330	Ishikawa (1999)
Ethiopia	subsistence agriculture, animal husbandry, semi-arid to humid	44	Fentahun & Hager (2009)
Ethiopia	country-wide estimate	203	Asfaw & Tadesse (2001)
Ethiopia	country-wide estimate	300	Asfaw (2009)
Ethiopia	agricultural, arid, open woodland (50% of plants in region edible)	25	Becker (1983)
Ethiopia	humid to semi-arid; forest to savannah, three ethnic groups in south Ethiopia	66	Balemie & Kebebew (2006)
Kenya	country-wide estimate for agricultural communities (plants only)	800	Maundu (1996)
Kenya	Turkana agro-pastoralists and rural fishing communities, arid and semi-arid	14	Levine & Crosskey (2006)
Madagascar	forest-dwelling, swidden cultivation in tropical forest	150	Styger *et al*. (1999)
Namibia	agriculture and livestock; tropical wetland, swamp and woodland in Caprivi	21	Mulonga (2003)
Nigeria	agricultural, savanna, semi-arid	121	Harris & Mohammed (2003)
Tanzania	agricultural, tropical forest, East Usambara mountains	28	Kessey (1998)
Tanzania	agricultural, tropical forest, East Usambara mountains	46	Hårkönen & Vainio-Mattila (1998)
Tanzania	Batemi agropastoralists, semi-arid (with 35 wild species cultivated)	44	Johns *et al*. (1996)
Uganda	agricultural households in southwest Uganda (some wild species cultivated and gathered from the wild)	94	Musinguzi *et al*. (2006)
Zambia	country-wide estimate	15–25	Pegler & Piearce (1980)

**Table 5.** The diversity of species of wild foods used by 12 indigenous communities (adapted from Kuhnlein *et al*. 2009)^a^.

no.	study area		ecosystem	flora	fauna	total species used
	cultural group	region				
1	Awajan	Peruvian Amazon	tropical forest	93	113	206
2	Bhil	Gujarat, India	tropical forest	68	23	91
3	Dalit	Andhra Pradesh, India	semi-arid	179	40	212
4	Karen	Thungyai Naresuan National Wildlife Sanctuary, Thailand	tropical; paddy cultivation	252	63	315
5	Mand (Pohnpei)	Pacific Ocean, Federated States of Micronesia	tropical	67	162	229
6	Igbo	Southern Nigeria	tropical	171	45	216
7	Ingano	Colombian Amazon	tropical forest	*x*^b^	92	(92 + *x*)
8	Ainu	Saru River Valley, Japan	riverine	10	3	13
9	Maasai	Kajiado District, Kenya	semi-arid	33	21	54
10	Inuit	Canadian Territory of Nunavut	polar	15	64	79
11	Nuxalk	Bella Coola, British Columbia	polar	42	25	67
12	Tetlit Gwich'in	Canadian Arctic	polar	15	35	50

^a^Communities 1–7 are formally seen as farming communities.

^b^Total cannot be accurately ascertained from original text as named traditional species are a mix of wild and cultivated.

Wild foods are still used in industrialized countries, though both use and traditional ecological knowledge appear to be declining (Mabey 1996; Pilgrim *et al*. 2008). In New Zealand, however, more than 60 species are still in common use, largely because of traditions of Maori groups. These include muttonbird (sooty shearwater), seagull, possum, rabbit, deer, wild pig, goat, salmon, trout, eel, watercress, sea lettuce, gorse and many berries (Newman & Moller 2005; NZFSA 2007; Stephenson & Moller 2009). In the Wallis Lake catchment, Australia, 88 species are in general use (Gray *et al*. 2005). In the swamps of Louisiana, large numbers of people still hunt and fish regularly for their own food (Roland 2006).

### (b) The nutritional value of wild foods

Malnutrition is a major health burden in developing countries, and the recognition that nutritional security and biodiversity are linked is fundamental for enlisting policy support to secure wild food use and preserve habitats for wild edible species. Comprehensive food composition data is a critical first step (McBurney *et al*. 2004; Flyman & Afolayan 2006; Frison *et al*. 2006). This is especially important for communities most vulnerable to malnutrition (Misra *et al*. 2008; Afolayan & Jimoh 2009). However, understanding of wild foods' micro- and macro-nutritional properties currently lags behind that of cultivated species (Vincetti *et al*. 2008).

Though several studies have found that wild foods are important sources of micronutrients, their energy-density is generally low (with the exception of honey and high-fat organs or in-season fat deposits) (Samson & Pretty 2006; McMichael *et al*. 2007). In the Sahel, several edible desert plants are sources of essential fatty acids, iron, zinc and calcium (Glew *et al*. 1997). In the arid Ferlo region of Senegal, some 50 per cent of all plants have edible parts, and those that are commonly consumed are critical suppliers of vitamins A, B2 and C, especially during seasonal lean periods (Becker 1983). Lockett *et al*. (2000) found that among the plants used by the Fulani in Nigeria, those available during the dry season (and thus important for ensuring year-round nutritional security in the face of possible food shortages) were superior in energy and micronutrient content compared with those from the wet season.

The contribution of dietary energy from traditional food species in 12 indigenous communities has been found to range from 30% to 93% of total dietary energy (Kuhnlein *et al*. 2009). For many indigenous communities, especially Artic and sub-Arctic, traditional wild foods outweigh modern store-bought items in terms of nutrient content. Their gradual replacement by store-bought produce causes discernable and significantly negative impacts on nutritional security at household and community levels (Samson & Pretty 2006).

## 4. The economic value of wild foods

### (a) Aggregate values

There is no comprehensive global estimate of the economic value of wild foods. Quantitative analyses face methodological difficulties. First, case studies using different valuation methods and diverse scales are rarely comparable. Second, sale of wild products (particularly bushmeat) is often illegal, and therefore under-reported. Trade is often informal or occurs at local markets and is therefore missed by conventional accounting mechanisms (Jaarsveld *et al*. 2005). The MEA (2005) cautions that the extent of freshwater fish catches might be under-reported by up to a factor of two because of inaccurate measures of informal fisheries.

While exact estimates of the economic value or volumes involved is difficult, what is not in dispute is that trade in and use of wild foods provide an important supplement to general incomes and are especially critical during economic hardship. Among the Tsimane' of Bolivia, only 3 per cent of goods consumed in the household comes from the market; a significant proportion comes from freshwater and forest (Reyes-García *et al*. 2008). In DR Congo, almost 90 per cent of harvested bushmeat and fish is sold rather than consumed (de Merode *et al*. 2003). In table 6, we summarize findings from economic valuations of direct use values for wild foods in selected African countries. From the limited data available, it is clear that wild plants and animals can provide $170–900 worth of value to rural households in South Africa and Tanzania. In Ghana, the bushmeat market is worth $275 million annually.

**Table 6.** Direct use values of wild foods valued either as contributions to household consumption or income from sale (selected African countries; 1999–2009). Note: GDP *per capita* (2009) figures in US$ (IMF 2009): DR Congo $171; Ghana $639; Namibia $4341; South Africa $5635; Tanzania $547; Zambia $1027.

country	consumption value within household	sale value (in US$)	reference
DR Congo	bushmeat 10%, fish 16%, wild plants 6%	n.a.	de Merode *et al*. (2003)
DR Congo	3–10% of total value of food consumed in the household	25% of all household sales	de Merode *et al*. (2003)
Ghana, country-wide estimate	n.a.	305 000 tonnes wild meat sold annually (value US$275 million)	IIED & Traffic (2002)
Namibia	21% respondents reported bushmeat cheaper than raised meat	wild plants value not recorded; fish: N$350 wk^−1^	Mulonga (2003)
South Africa	wild foods comprise 31% of all plants on residential plots, and 72% of the value of all plant products consumed	28% of all plant products sold: US$269 household^−1^yr^−1^, of which wild foods worth US$83	High & Shackleton (2000)
South Africa	R2819—R7238 household^−1^yr^−1^ (wild foods are a part)	US$367–941 household^−1^yr^−1^	Shackleton *et al*. (2002)
South Africa	$167 household^−1^yr^−1^	US$167 household^−1^yr^−1^	Dovie *et al*. (2006)
Tanzania	n.a.	58% of cash income from sale of NTFPs and wild foods	Makonda & Gillah (2007)
Tanzania		US$378 household^−1^yr^−1^: $265 plant-medicines; $15 wild vegetables; $27 wild fruit; $21 leaves and stems; $20 wild animals; $10 insects; $18–126 wild honey	Kasthala *et al*. (2008)
Zambia	n.a.	US$2.15 kg^−1^ in rural areas (three to four times more in urban areas)	Jumbe *et al*. (2008)
Zambia	n.a.	US$4 gallon^−1^; during season collectors can earn up to a month's salary for a general worker	Mbata *et al*. (2002)

### (b) Values to the poorest households

An important aspect of wild food use is the relative importance of wild foods to poorer households. The conventional understanding holds that poorer households depend more on wild foods. However, detailed analyses do not show simple correlations between wealth and resource use (de Merode *et al*. 2003; Allebone-Webb 2009). A range of context-specific social and economic factors (e.g. price, individual or cultural preference, and wealth) are also relevant.

In some countries, household consumption of wild foods increases with wealth—with the exception of bushmeat in Africa (IIED & Traffic 2002). de Merode (2003) found that the poorest households among those sampled in DR Congo were unable to capitalize on the most valuable food products and concluded that household use of wild foods depends less on natural abundance than on socio-economic factors. In Honduras, the sale of forest products as an emergency response was relatively restricted to a minority of households and only certain conditions of cash need. Most households preferred other short-term measures such as the sale of stored crops, borrowing cash or doing wage labour (McSweeney 2005). Consumption is also influenced by price or individual or cultural preference.

## 5. Drivers of change in wild food availability and use

There are a number of important drivers for wild food availability and use. While some clearly increase or decrease the use of wild foods, the impact of others is ambiguous and context-dependent. The importance of understanding current trends for wild foods is underscored by the recognition that food insecurity is a particular problem among indigenous populations (Ford & Berrang-Ford 2009). For instance, Willows *et al*. (2009) find that of 35 000 households, 1528 of whom were aboriginal, 33 per cent of Aboriginal households were food-insecure, compared with 9 per cent of non-Aboriginal households, and that Aboriginal households were more prone to experience socio-demographic risk factors for food insecurity than non-Aboriginal households. The *interaction* between drivers also deserves attention. In assessing links between local knowledge and sociocultural continuity, Howard (2010) finds that cultural identity and agrobiodiversity are strongly associated: ‘culture and ecosystems … co-evolve’. Thus, a biophysical driver (e.g. climate change) could have knock-on effects on a cultural parameter (e.g. local knowledge), and the effect of the two combined could lead to either an increase or decrease in wild food use.

### (a) Wild foods in a changing climate

Forecasting the precise impacts of the changing climate on the availability of wild foods is difficult (MEA 2005; Woodruff *et al*. 2006). Studying resilience and vulnerability in two communities in Tanzania and Niger, Strauch *et al*. (2009) concluded that there was insufficient evidence to predict the impacts that climate change would have on both human foraging and the interlinked processes of local ecological knowledge (LEK) transmission, cultural continuity and land-based subsistence livelihood.

At a regional level, White *et al*.'s (2007) study of the effects of a changing climate on wild food supplies in the Arctic focused on surface water regimes. There were multiple impacts brought by changes in hydrology for local communities. The stresses brought by a changed Arctic climate are compounded by rapid socio-cultural change in the region (Samson & Pretty 2006; Loring & Gerlach 2009). Wild food species offer a potentially critical role for buffering against food stress caused by a changing climate. Nevertheless, ‘the innate resilience of wild species to rapid climate change, which is often lacking in exotic species’, means that they could play an increasingly important role during periods of low agricultural productivity associated with climate events (Fentahun & Hager 2009).

### (b) Land use change and degradation

Current trends in land use, including expansion of intensive agriculture, limit the capacity of ecosystems to sustain food production and maintain the habitats of wild food species (Foley *et al*. 2005). Changes in land use and agriculture expansion have significant implications for the availability of wild foods. The commercialization of agriculture—an important driver of land use change—potentially implies decreased reliance on wild foods (Treweek *et al*. 2006). Agricultural and land use policy, infrastructure development and widened access to markets all drive land use change, and are implicated in declines of wild species in Thailand (Schmidt-Vogt 2001; Padoch *et al*. 2007) and China (Xu *et al*. 2009).

Biodiversity in intensely managed swidden (shifting) fallows has traditionally provided communities with the means to increase incomes, improve diets and increase labour productivity. Most of the wild food species used by swiddeners come from fallows, rather than mature forests. With the replacement of swidden farming by annual or perennial crops (Bruun *et al*. 2009), wild foods that accompanied fallows are being lost, leading to decreased diversity, and with it downgraded nutritional status, health and income, and the removal of a vital ‘safety net’ for the rural poor (Rerkasem *et al*. 2009). Somnasang *et al*. (1998) report that in 20 villages surveyed in Thailand, deforestation had led to a decline in wild food species. Efforts by the local community to stem this loss by domesticating important species were unsuccessful, as many species do not survive outside their natural forested habitat.

Overall, the challenge of feeding a growing world population, if it does not focus on sustainable intensification (Royal Society 2009), will further threaten naturally biodiverse landscapes. Yet, ensuring dietary diversity and associated nutritional security rests on ‘forestalling the imminent extinction of up to one quarter of the worlds' wild species and the loss of important agro-biodiversity’. This calls for a biodiversity-focused strategy in food, public health and poverty-alleviation policies (Johns & Sthapit 2004).

### (c) Unsustainable harvesting

Sixteen of the world's biodiversity hotspots correspond with areas of malnutrition and hunger, placing pressure on biodiversity for food provision (Treweek *et al*. 2006). In these locations, unsustainable harvests have led to declines in wild food species.

The illegal use and trade of bushmeat is well documented. In the long term, over-harvesting will have a negative impact on wild food availability and thus on nutritional security for those communities that rely on bushmeat for protein. In some parts of Africa, unsustainable harvesting is putting added pressure on stocks. An important driver is the widespread availability of firearms (Jaarsveld *et al*. 2005). Nevertheless, despite the fact that unsustainable trade in bushmeat is regarded as a threat to wildlife, Cowlishaw *et al*. (2005) found some evidence of sustainable harvesting after the extinction (through historical hunting) of key species. After vulnerable species have been depleted, robust species (fast reproducers) are then harvested and traded at sustainable levels. Management policies might therefore benefit from according stricter protection to key species but allowing robust ones to continue being traded sustainably.

Where species have traditionally been harvested sustainably, the entry of the market and the commercialization of species hitherto used exclusively for local subsistence can also result in over-harvesting (Kala 2009). Unsustainable harvesting is a concern in the case of wild fisheries. At a global level, increasing average *per capita* consumption of seafood has led to catch rates that regularly exceed maximum sustainable yields (MEA 2005). Brashares *et al*. (2004) found links between unsustainable harvesting of bushmeat and fish stocks in Africa: years of poor fish catches coincided with increased hunting over a 30 year period.

### (d) Deepening poverty, HIV*/*AIDS and conflict

In Africa, climate-induced vulnerabilities combined with HIV/AIDS have produced a decline in food security sufficiently great to have spurred new thinking on the origins of famine (e.g. New Variant Famine Hypothesis: de Waal & Whiteside 2003). Hlanze *et al*. (2005) state that ‘increasingly it is becoming difficult to separate the food security impact of drought from that of HIV/AIDS. The two work in tandem to cause poor harvests and reduced incomes.’ For households afflicted by HIV/AIDS, wild foods offer nutritious dietary supplements at low labour and financial costs. This is important when considering the negative impact of a household's HIV/AIDS status on income and food security (Kaschula 2008), together with the fact that deficiencies of micronutrients (in which many wild foods are rich) critical to immune-system function are ‘commonly observed in people living with HIV in all settings’ (Piwoz & Bentley 2005). Food stress associated with HIV/AIDS can drive households to intensify wild food use, putting unsustainable pressure on local resources especially when combined with deepening poverty or indeed conflict (Dudley *et al*. 2002). In South Africa, Kaschula (2008) found that wild food use was significantly more likely in households afflicted by HIV.

However, use of wild foods could also *decline* due to HIV/AIDS. For example, at one site, it was found that ‘households suffering the loss of a head of household were actually less likely to gather from the bush’ (Hunter *et al*. 2009). Further relevant drivers include the loss of ecological knowledge as adults die (Ansell *et al*. 2009), declines in household labour (de Waal & Whiteside 2003; Kaschula 2008) and the stigma attached to HIV/AIDS (Kaschula 2008).

Armed conflict and associated internal displacement of populations are associated with heavy subsistence use of wild foods by refugees, combatants and resident non-combatants alike, and the sale or barter of wildlife for food (Loucks *et al*. 2009), arms or other goods. Conflict—often positively correlated with areas of high biodiversity—is generally associated with landscape degradation (Loucks *et al*. 2009). It is conceivable that this could lead to a decline in the long-term use of wild food species. Climate change is also predicted to increase armed conflict in some developing countries (Buhaug *et al*. 2008).

### (e) Loss of local ecological knowledge

LEK is required for the identification, collection and preparation of wild foods (Pilgrim *et al*. 2008). The distribution of LEK between individuals in a community is usually differentiated by gender, age or social role. Several studies show women score higher on food-related knowledge (Price 1997; Somnasang *et al*. 1998; Styger *et al*. 1999). In one Nepalese site, women above 35 years of age were able to describe the uses of 65 per cent of all edible species, while young men could only describe 23 per cent (Shrestha & Dhillon 2006). Men and women might also hold specialized LEK. Somnasang *et al*. (1998) found that while men had more knowledge of hunting and fishing, women had more knowledge of wild food plants, insects and shrimp. LEK is also differentiated by age: in Ethiopia, children gather fruit for consumption by the whole community, and unsurprisingly those under 30 had the most knowledge of wild fruits (Fentahun & Hager 2009).

Research has pointed to declines in LEK (Pilgrim *et al*. 2008) as communities rely increasingly on store-bought foods and move away from land-based livelihoods. Somnasang *et al*. (1998) found that young people working outside the village did not have the chance, and in some cases the desire, to acquire food-relevant LEK. It is thus possible that as young adults leave land-based livelihoods, knowledge transmission to younger generations will be diminished. In other cases, individuals' preferences change as they grow and thus, their stock of LEK changes, even if they remain within their community. In Ethiopia, Fentahun & Hager (2009) found that ‘… grown-ups succumb to the culture of the society which regards the consumption of wild fruits (commonly consumed by children) as a source of shame’ (insert added). As climate change alters habitats, so knock-on effects are expected on LEK (Strauch *et al*. 2009).

### (f) Socio-economic change and the expansion of markets

The nutrition transition associated with industrialization and the modernization of diets poses challenges to public health worldwide (Popkin 1998). The replacement of wild foods by store-bought products is linked to reduced dietary diversity, rising rates of chronic lifestyle-related conditions such as obesity and type II diabetes, poor intake of micronutrients (Batal & Hunter 2007) and malnutrition (Erikson *et al*. 2008). Traditional species become undervalued and underused as exotic ones become available, as has been found in India (Rathore 2009) and the Amazon (Byron 2003). Yet, the importance of wild foods to *nutritional* security means that they are not necessarily replaced by store-bought foods providing the same amount of calories. Global trends indicate that more people will, however, come to depend solely on store-bought, cultivated foods (Johns & Maundu 2006), thus marginalizing wild foods.

In regions isolated from sweeping transformations, traditional food systems can persist. Pieroni (1999) suggests that the geographical isolation of the upper Serchio valley in northwest Tuscany has ‘permitted a rich popular knowledge to be maintained’. Gastronomic traditions in the valley help to preserve influences dating from pre-Roman times and over 120 species form a well-preserved pharmacopoeia of food and medicine. In other regions too, wild food use seems to persist: 123 edible species are still used in Spain (Tardío *et al*. 2003); and in many Mediterranean countries, wild foods are still prevalent enough to be considered an important part of local diets (Leonti *et al*. 2006).

In the Arctic, the nutrition transition is driven by a changing climate as well as large-scale cultural changes. This transition produces significant negative effects to physical and mental health at community level (Samson & Pretty 2006; Loring & Gerlach 2009). In the Canadian Artic, children now obtain more than 40 per cent of their total energy from store-bought processed foods (‘sweet’ and ‘fat’ foods). In adults, however, the benefits of consuming traditional wild foods are clear: ‘… even a single portion of local animal or fish food resulted in increased (*p* < 0.05) levels of energy, protein, vitamin D, vitamin E, riboflavin, vitamin B-6, iron, zinc, copper, magnesium, manganese, phosphorus, and potassium’ (Kuhnlein & Receveur 2007). Though wild foods have traditionally played a critical role in circumpolar communities (Ford 2009; Ford *et al*. 2009; Titus *et al*. 2009), public health policy across many countries tends to operate within a model of food security that discounts the traditional food practices of these communities (Power 2008).

## 6. Securing the future for wild foods

The MEA (2005) lists 250 mammalian, 262 avian and 79 amphibian species as threatened from overexploitation for food. Mechanisms such as CITES regulate cross-border trade in wild species, but require international cooperation. At national level, however, trade is generally poorly regulated and monitored. Challenges to sustainable harvesting include (i) lack of comprehensive data on species used and sustainable yields; (ii) lack of management regimes and institutions regulating ownership, access and harvesting rights; (iii) lack of legislation and policy for sustainable harvesting—in many cases a result of lack of information on use and trade of species (Schippmann *et al*. 2006).

Policy support is central to the conservation of species as well as LEK. Lack of policy support for relevant programmes has been implicated in the continued over-harvesting of African bushmeat (Scholes & Biggs 2005). By contrast, support for agroforestry systems have potentially ensured sustainable harvests from indigenous tree species in areas otherwise prone to deforestation (Sileshi *et al*. 2007). Management of common forests has recently become successful with the emergence of joint forest management and community-managed forest groups (Ostrom *et al*. 2002; Pretty 2003; Berkes 2004; Molnar *et al*. 2007). Worldwide, some 370 million ha of various habitats are estimated to be under community conservation, including 14 million ha managed by 65 000 community groups in India and 900 000 ha managed by 12 000 groups in Nepal. In Italy, Vitalini *et al*. (2009) linked the continued use of wild food and plants with a site's EU designation of ‘Site of Community Interest’. The preservation of habitats bodes well for species conservation, but there are also concerns that protected area status might exclude local people from access and use.

In environments where LEK is being lost, it is important that it be recorded. Local communities might themselves desire to preserve wild food species through, for example, the establishment of community enterprises based on wild food resources in Nepal (Shrestha & Dhillon 2006) or through local women strengthening traditional community sanctions against overuse and enlisting the support of state law in northeast Thailand (Price 1997).

## 7. Conclusions

Wild food species form a significant portion of the total food basket for households from agricultural, hunter, gatherer and forager systems. However, the focus on the contribution of agriculture to total food security has resulted in the routine undervaluation of wild food species. The continued contribution of wild species to food and nutritional security is threatened by some of the processes that seek to increase agricultural production and enhance economic development. While wild foods cannot entirely bridge the existing supply and demand gaps, without them it would be much wider.

Edible species provide more than just food and income. In communities with a tradition of wild food use, it is part of a living link with the land, a keystone of culture (Pretty 2007; Pilgrim & Pretty 2010). The decline of traditional ways of life and decreased wild food use are interlinked. Research needs are twofold: (i) standardized, accessible and comparable studies on the nutritional and toxicological properties of currently underused wild species on a broad scale; (ii) the identification of priority areas for conservation of wild food species and the recording of food-relevant LEK. Polices on conservation, food-security and agriculture need to be integrated to recognize and preserve the importance of wild foods.

Recent initiatives indicate that this may be taking place. For example, traditional food revitalization projects aim to increase the consumption of wild foods, and are being used to provide health and cultural benefits to traditional communities otherwise subject to the nutrition transition (Pilgrim *et al*. 2009). The FAO recognizes that ‘nutrition and biodiversity converge to a common path leading to food security and sustainable development’ and that ‘wild species and intraspecies biodiversity have key roles in global nutrition security’ (FAO 2009). The evidence shows that wild foods provide substantial health and economic benefits to those who depend on them. It is now clear that efforts to conserve biodiversity and preserve traditional food systems and farming practices need to be combined and enhanced.
